# Diverse allosteric and catalytic functions of tetrameric d-lactate dehydrogenases from three Gram-negative bacteria

**DOI:** 10.1186/s13568-014-0076-1

**Published:** 2014-10-28

**Authors:** Nayuta Furukawa, Akimasa Miyanaga, Misato Togawa, Masahiro Nakajima, Hayao Taguchi

**Affiliations:** 1 Tokyo University of Science, 2641 Yamazaki, Noda 278-8510, Chiba, Japan; 2 Tokyo Institute of Technology, 2-12-1 O-okayama, Meguro-ku 152-8551, Tokyo, Japan

**Keywords:** Allosteric regulation, NAD-dependent d-lactate dehydrogenase, Gram-negative bacteria, Escherichia coli, Fusobacterium nucleatum, Pseudomonas aeruginosa

## Abstract

NAD-dependent d-lactate dehydrogenases (d-LDHs) reduce pyruvate into d-lactate with oxidation of NADH into NAD^+^. Although non-allosteric d-LDHs from *Lactobacilli* have been extensively studied, the catalytic properties of allosteric d-LDHs from Gram-negative bacteria except for *Escherichia coli* remain unknown. We characterized the catalytic properties of d-LDHs from three Gram-negative bacteria, *Fusobacterium nucleatum* (FNLDH), *Pseudomonas aeruginosa* (PALDH), and *E. coli* (ECLDH) to gain an insight into allosteric mechanism of d-LDHs. While PALDH and ECLDH exhibited narrow substrate specificities toward pyruvate like usual d-LDHs, FNLDH exhibited a broad substrate specificity toward hydrophobic 2-ketoacids such as 2-ketobutyrate and 2-ketovalerate, the former of which gave a 2-fold higher *k*_cat_/S_0.5_ value than pyruvate. Whereas the three enzymes consistently showed hyperbolic shaped pyruvate saturation curves below pH 6.5, FNLDH and ECLDH, and PALDH showed marked positive and negative cooperativity, respectively, in the pyruvate saturation curves above pH 7.5. Oxamate inhibited the catalytic reactions of FNLDH competitively with pyruvate, and the PALDH reaction in a mixed manner at pH 7.0, but markedly enhanced the reactions of the two enzymes at low concentration through canceling of the apparent homotropic cooperativity at pH 8.0, although it constantly inhibited the ECLDH reaction. Fructose 1,6-bisphosphate and certain divalent metal ions such as Mg^2+^ also markedly enhanced the reactions of FNLDH and PALDH, but none of them enhanced the reaction of ECLDH. Thus, our study demonstrates that bacterial d-LDHs have highly divergent allosteric and catalytic properties.

## 1 Introduction

NAD-dependent d- and l-lactate dehydrogenases (d-LDH, EC 1.1.1.28; and l-LDH, EC 1.1.1.27) reduce pyruvate to d- and l-lactate, respectively, with oxidization of NADH into NAD^+^, and act at the last step of the glycolytic pathway under anaerobic conditions (Holbrook et al. [1975]). Although both types of enzymes catalyze identical reactions except for the distinct chirality of the lactate product, they are evolutionarily distinct from each other (Taguchi and Ohta [1991]; Bernard et al. [1991]; Kochhar et al. [1992]). d-LDHs belong to a large d-2-hydroxyacid dehydrogenase (d-HydDH) superfamily, which comprises various kinds of d-isomer dehydrogenases such as the d-glycerate (Greenler et al. [1989]; Goldberg et al. [1994]), d-3-phosphoglycerate (Tobey and Grant [1986]; Grant [1989]), d-2-hydroxyglutarate (Buckel and Barker [1974]; Martins et al. [2005]), and d-2-hydroxyisocaproate (Lerch et al. [1989]; Dengler et al. [1997]) dehydrogenases, and even non- d-HydDHs such as the formate (Vinals et al. [1993]; Lamzin et al. [1994]; Popov and Lamzin [1994]), phosphite (Costas et al. [2001]; Woodyer et al. [2005]), and l-alanine (Baker et al. [1998]; Tripathi and Ramachandran [2008]) dehydrogenases. Although these enzymes possess highly divergent primary sequences, their tertiary structures are very similar to one another.

In the case of l-LDHs, it is known that allosteric types of the enzyme are widely distributed in bacteria including *Lactobacilli* (Garvie [1980]). These enzymes commonly require fructose 1,6-bisphosphate (FBP) to exhibit high catalytic activity, and usually exhibit sigmoidal shaped substrate saturation curves unless FBP is present. l-LDHs generally have tetrameric structures comprising four identical subunits, and allosteric l-LDHs undergo cooperative structural changes in the substrate and FBP binding sites through quaternary structural changes (Iwata et al. [1994]; Arai et al. [2010]). On the other hand, no allosteric property has been reported for *Lactobacillus*d-LDHs. The *Lactobacillus*d-LDHs consistently have a dimeric structure comprising identical subunits, and exhibit hyperbolic shaped saturation curves for substrate pyruvate. Interestingly, it was reported that d-LDH from *E. coli* exhibits a sigmoidal pyruvate saturation curve, which is changed to the hyperbolic one by 2-ketobutyrate (Tarmy and Kaplan [1968]). The reported results of size-exclusion chromatography suggested that this enzyme has a homotetrameric structure, although the detailed catalytic properties and 3D structure of the enzyme remain unknown. Beside the d-LDH from *E. coli,* the d-3-phosphoglycerate dehydrogenases (d-PgDHs) from some bacteria, such as *E. coli* (Tobey and Grant [1986]; Grant [1989]; Schuller et al. [1995]) and *Mycobacterium tuberculosis* (Dey et al. [2005]), are only known as allosteric and homotetrameric enzymes in the d-HydDH superfamily.

In this paper, we describe the detailed biochemical analysis of allosteric d-LDHs from three Gram-negative bacteria; *Fusobacterium nucleatum* subsp. *nucleatum*, an obligate anaerobe; *Pseudomonas aeruginosa*, an obligate aerobe; and *E. coli*, a facultative anaerobe, demonstrating that bacterial d-LDHs have highly divergent catalytic properties.

## 2 Materials and methods

### 2.1 Cloning, expression, and purification

Genomic DNA of *F. nucleatum* subsp. *nucleatum* JCM14847 (=ATCC25586) and *P. aeruginosa* JCM8532 (=PAO1) was purchased from the Riken BioResource Center (Japan). Genomic DNA of *E. coli* BL21(DE3) was isolated by ISOGEN (NIPPON GENE, Tokyo, Japan). The genes encoding the FN0511 (FNLDH), PA0927 (PALDH), and ECD_01352 (ECLDH) proteins (Genbank accession numbers: AAL94707.1, AAG04316.1, and ACT43236.1, respectively) were amplified *via* PCR using genomic DNA as templates. The primer pairs used were, 5′-CGCTCGAGATGCAAAAAACTAAGATAATATTTTTTG-3′ and 5′-GGGATCCATTTATTGATTTTGTGGAACTTC-3′ for the *fn0511* gene, 5′-CGCATATGCGCATCCTGTTCTTCAGCAG-3′ and 5′- CGGGATCCTCAGGCCCGGACCCGATTG-3′ for the *pa0927* gene, and 5′-CGCATATGAAACTCGCCGTTTATAGC-3′ and 5′-CGGGATCCTTAAACCAGTTCGTTCGGGC-3′ for the *ecd_01352* gene. The amplified *fn0511*, *pa0927*, and *ecd_01352* genes were inserted into T-vector pMD20 (TaKaRa Bio, Shiga, Japan). After purification of the plasmids carrying these genes, the plasmids were digested with restriction enzymes and the genes were inserted into pCold I (TaKaRa Bio). *E. coli* Rosetta2 (DE3) (Merck Millipore, Darmstadt, Germany) was transformed using the constructed plasmids and cultured at 37°C in 2 × YT medium (1.6% tryptone, 1.0% yeast extract and 0.5% NaCl) containing 100 μg/ml ampicillin. After the optical density of the culture at 600 nm (OD_600_) had reached 0.6, protein expression was induced using 0.5 mM isopropyl-β-1-d-thiogalactopyranoside at 15°C overnight. The harvested cells were lysed by sonication in 50 mM HEPES-NaOH buffer (pH 8.0) containing 150 mM NaCl and 10 mM imidazole on ice. After each sample had been centrifuged at 27,000 × *g*, the supernatant was applied to HisTrap FF crude column (5 ml) (GE Healthcare, Buckinghamshire, UK), and was then eluted with a linear gradient of 10–250 mM imidazole. After dialysis against 50 mM HEPES-NaOH buffer (pH 8.0), the protein solution was further purified using a UnoQ column (Bio-Rad Laboratories, California, USA). The target protein was eluted with a linear gradient of 0–300 mM NaCl. The buffer for FNLDH and ECLDH was changed to 5 mM HEPES-NaOH buffer (pH 8.0), and the buffer for PALDH to 5 mM sodium acetate buffer (pH 5.0) using Amicon Ultra 30,000 molecular weight cut-off (Merck Millipore). The concentrations of FNLDH, PALDH, and ECLDH were determined spectrophotometrically at 280 nm using theoretical extinction coefficients of 24,215, 19,285, and 17,140 M^−1^ cm^−1^, respectively (Pace et al. [1995]).

### 2.2 SDS-PAGE

The pufiried enzymes were separated on a 11% (w/v) SDS-polyacrylamide gel (Laemmli [1970]). Approximately 5 μg of the enzymes were loaded as 5% (v/v) glycerol solutions containing 1% (w/v) SDS, 2.5% (v/v) β-mercaptoethanol and 0.05% bromophenol blue. Precision Plus Protein Unstained Standards (Bio-Rad Laboratories) was used as molecular weight markers. Proteins were stained with Coomassie Brilliant Blue.

### 2.3 Enzyme assay

Enzyme activity was determined by measuring the reduction of the absorbance at 340 nm derived from NADH for 1 minute. The reactions were performed at 30°C in various 50 mM buffers containing 0.1 mM NADH and a substrate. The data that showed significant cooperative effects of the substrate were interpreted using the Hill equation (Equation 1) (Dixon and Webb [1979]).

(1) $$\left(v-v_{\min}\right)/k_{\mathrm{cat}}={{\left[S\right]}^{n}}^{H}/\left({{\left[S\right]}^{n}}^{H}+S_{0.5}{{}^{n}}^{H}\right)$$

where *v* is the reaction velocity, [S] the ligand concentration, such as pyruvate, FBP, or Mg^2+^, *k*_cat_ the turnover rate of catalysis, *v*_min_ the reaction velocity with no ligand, *S*_0.5_ the half-saturation concentration of a ligand, and *n*_H_ the Hill coefficient. Kinetic parameters were obtained by curve fitting of the data with KaleidaGraph ver 3.51. The data that indicated significant substrate inhibition were interpreted using the equation for substrate inhibition (Equation 2) (Eszes et al. [1996]).

(2) $$v/k_{\mathrm{cat}}=\left[S\right]/\left(\left[S\right]+S_{0.5}+{\left[S\right]}^{2}/K_{I}\right)$$

where *K*_i_ is the inhibition constant. The kinetic parameters for oxamate at pH 7.0 were calculated by curve fitting of the data with GraFit version 7.0.3. The data that indicated significant competitive-type and mix-type inhibition were interpreted using the equation for competitive-type (Equation 3) and mix-type inhibition (Equation 4), respectively.

(3) $$v/k_{\mathrm{cat}}=\left[S\right]/\left(K_{m}\left(1+\left[I\right]/K_{I}\right)+\left[S\right]\right)$$

(4) $$v/k_{\mathrm{cat}}=\left[S\right]/(K_{m}\left(1+\left[I\right]/K_{I}\right)+\left[S\right]\left(1+\left[I\right]/K_{I}’\right)$$

where *K*_m_ is the Michaelis constant, and *K*_i_’ the inhibition constant. The data of oxamate inhibition for FNLDH, PALDH, and ECLDH at pH 8.0 were interpreted using the equation for competitive-type (Equation 5), mix-type (Equation 6), and non-competitive-type (Equation 7) inhibition of allosteric enzyme, respectively.

(5) $$v={{\left[S\right]}^{n}}^{H}/\left(K_{m}{{}^{n}}^{H}\left(1+\left[I\right]/K_{I}\right)+{{\left[S\right]}^{n}}^{H}\right)\times \left(k_{\mathrm{cat}}+k_{\mathrm{cat}}’{{\left[I\right]}^{n}}^{H’}/\left({{\left[I\right]}^{n}}^{H’}+K_{\mathrm{act}}{{}^{n}}^{H’}\right)\right)$$

(6) $$v={{\left[S\right]}^{n}}^{H}/\left(K_{m}{{}^{n}}^{H}\left(1+\left[I\right]/K_{I}\right)+{{\left[S\right]}^{n}}^{H}\left(1+\left[I\right]/K_{I}’\right)\right)\times \left(k_{\mathrm{cat}}+k_{\mathrm{cat}}’{{\left[I\right]}^{n}}^{H’}/\left({{\left[I\right]}^{n}}^{H’}+K_{\mathrm{act}}{{}^{n}}^{H’}\right)\right)$$

(7) $$v=k_{\mathrm{cat}}{{\left[S\right]}^{n}}^{H}/\left(\left(K_{m}{{}^{n}}^{H}+{{\left[S\right]}^{n}}^{H}\right)\left(1+\left[I\right]/K_{I}\right)\right)$$

where *k*_cat_’ is the turnover rate of catalysis in the presence of oxamate, *K*_act_ the half-saturation concentration of oxamate for activation, and *n*_H_’ the hill coefficient for oxamate.

### 2.4 Temperature and pH stability

The pH-stability was determined by measuring the remaining activity after incubation of the enzymes (1 μM) in various 50 mM buffers at 30°C for 1 h. The thermostability was determined by measuring the remaining activity after incubation of FNLDH (1 μM) or ECLDH (1 μM) in 50 mM HEPES-NaOH buffer (pH 8.0), or of PALDH (1 μM) in 50 mM sodium acetate buffer (pH 5.0) at various temperatures for 30 min.

### 2.5 Size-exclusion chromatography

Each protein solution diluted to 1 mg/ml (1 ml) was applied to Superdex 200 (Hiload 16/60; GE Healthcare) equilibrated with 50 mM buffer containing 150 mM NaCl. HEPES-NaOH (pH 8.0) was used for FNLDH and ECLDH, and sodium acetate buffer (pH 5.0) for PALDH. Ovalbumin (44 kDa), conalbumin (75 kDa), aldolase (158 kDa), ferritin (440 kDa), and thyroglobulin (669 kDa) (GE Healthcare) were used as standard proteins. Blue dextran 2000 (2,000 kDa; GE Healthcare) was used to determine the void volume of the column.

## 3 Results

### 3.1 Basic properties

**Figure 1:**
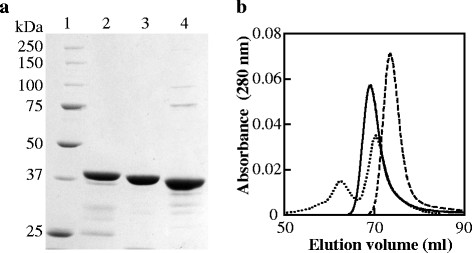
**SDS-PAGE and size-exclusion chromatography of the purified enzymes. a)** Lane 1, molecular mass standards (kDa); lane 2, FNLDH; lane 3, PALDH; lane 4, ECLDH. **b)** Elution of the enzymes from Superdex 200 with the buffers described in ‘Materials and methods’. Solid, dashed, and dotted lines indicate FNLDH, PALDH, and ECLDH, respectively.

**Figure 2:**
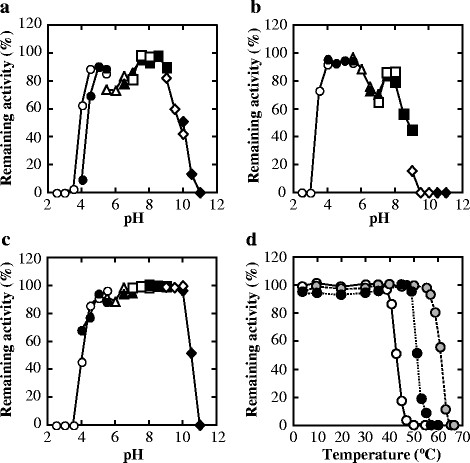
**pH-stability of FNLDH (a), PALDH (b), and ECLDH (c), and heat-stability of the enzymes (d). a-c)** Each enzyme was treated at 30°C for 1 h in sodium citrate (open circles), sodium acetate (closed circles), MES-NaOH (open triangles), MOPS-NaOH (closed triangles), HEPES-NaOH (open squares), Bicine-NaOH (*N,N*-Bis(2-hydroxyethyl)glycine) (closed squares), CHES-NaOH (*N*-cyclohexyl-2-aminoethanesulfonic acid) (open diamonds), and CAPS-NaOH (*N*-cyclohexyl-3-aminopropanesulfonic acid) (closed diamonds) buffers. **d)** FNLDH (white circles and solid lines), PALDH (grey circles and dashed lines), and ECLDH (black circles and dotted lines) were treated at various temperatures for 30 min in the buffers described in ‘Materials and methods’.

The recombinant FNLDH, PALDH and ECLDH consistently exhibited marked catalytic activity toward pyruvate, and were successively purified to homogeneous protein samples (Figure 1a). FNLDH, PALDH, and ECLDH were stable in the pH ranges of 5.0–8.5, 4.0–8.0, and 5.0–10.0, respectively, during treatment at 30°C for 1 h (Figure 2 a, b, c). FNLDH, PALDH, and ECLDH were stable up to 39, 57, and 49°C, respectively, under favorable pH conditions, i.e., pH 8.0 for FNLDH and ECLDH, and pH 5.0 for PALDH (Figure 2d).

The purified FNLDH and PALDH samples each gave a single protein peak on size-exclusion chromatography analysis, and exhibited apparent molecular weights of 160 and 120 kDa, respectively (Figure 1b). On the other hand, the ECLDH sample gave two peaks corresponding to molecular weights of 330 and 150 kDa. The theoretical molecular weights of FNLDH, PALDH, and ECLDH are 40.0, 37.8, and 38.5 kDa, suggesting that FNLDH and PALDH at that of homotetrameric and homotrimeric structures, respectively, and ECLDH was eluted at the position of homooctameric and homotetrameric structures. Since it has been reported that native d-LDH from *E. coli* cells gives only a peak that corresponds to the molecular weight of 130 kDa (Tarmy and Kaplan [1968]), the apparently octameric form of ECLDH is likely an artificial product due to the overexpression, and exhibited slightly but significantly lower specific activity than the tetrameric form (data not shown). Therefore, only homotetrameric ECLDH was used for the detailed analysis below.

### 3.2 pH-Dependency of kinetic parameters

**Figure 3:**
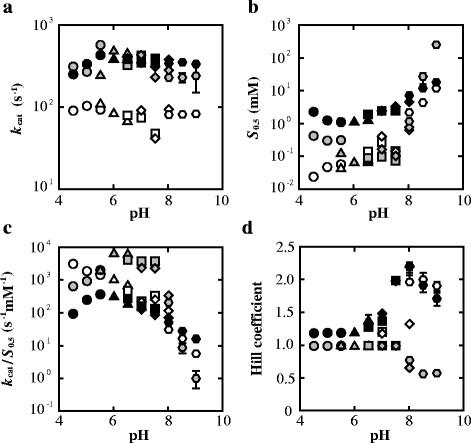
**pH dependence of kinetic parameters on pyruvate reduction.** White, grey, and black circles indicate FNLDH, PALDH, and ECLDH, respectively. The *k*_cat_**(a)**, *S*_0.5_**(b)**, and *k*_cat_/*S*_0.5_ data **(c)** are plotted logarithmically, and the Hill coefficient **(d)** is plotted linearly. The buffers used for the assay were sodium acetate buffer (pH 4.5, 5.0, and 5.5; circles), MES-NaOH buffer (pH 5.5, 6.0, and 6.5; triangles), MOPS-NaOH buffer (pH 6.5, 7.0, and 7.5; squares), HEPES-NaOH buffer (pH 7.0, 7.5 and 8.0; diamonds), and Bicine-NaOH buffer (pH 8.0, 8.5, and 9.0; hexagons). We adjusted the pH of each buffer solution prior to the addition of substrate and cofactor, and confirmed that the pH value is not affected even in the presence of high concentrations of substrate.

The kinetic parameters, *k*_cat_, *S*_0.5_, *k*_cat_/*S*_0.5_, and *n*_H_ values, were determined by pyruvate reduction assaying in the pH range of 4.5-9.0 (Figure 3). The three enzymes consistently showed virtually constant *k*_cat_ values independently of pH, and PALDH and ECLDH exhibited about 5-fold larger *k*_cat_ values than FNLDH. The three enzymes also consistently showed constant pyruvate *S*_0.5_ values below pH 7.0, and PALDH and FNLDH exhibited approximately one order of magnitude smaller *S*_0.5_ values than ECLDH. Above pH 8.0, the three enzymes exhibited increased *S*_0.5_ values, depending on the pH, and their *k*_cat_/*S*_0.5_ values changed mostly according to the changes in the *S*_0.5_ values. ECLDH apparently showed lower pH-dependence of the *S*_0.5_ value than the other two enzymes, of which PALDH showed slightly higher pH-dependence than FNLDH. Under acidic conditions below pH 6.0, the three enzymes consistently showed hyperbolic shaped pyruvate saturation curves, and no marked cooperativity in pyruvate binding. Under higher pH conditions, however, FNLDH and ECLDH showed markedly sigmoidal saturation curves for pyruvate, i.e., positive homotropic cooperativity, giving maximal *n*_H_ values of about 2.0. In contrast, PALDH showed negative homotropic cooperativitiy above pH 8.0, giving a minimal *n*_H_ value of 0.5.

### 3.3 Substrate specificity

The kinetic parameters of the d-LDHs for various 2-ketoacids are summarized in Table 1. For pyruvate, PALDH exhibited the highest catalytic efficiency among the three enzymes, since it exhibited a small *S*_0.5_ like FNLDH and a large *k*_cat_ like ECLDH. ECLDH showed a narrow substrate specificity toward pyruvate among hydrophobic 2-ketoacid substrates, although ECLDH consistently exhibited relatively high catalytic activity toward oxaloacetate, glyoxylate and hydroxypyruvate. PALDH showed a similar substrate specificity to that of ECLDH, exhibiting relatively high catalytic activity toward oxaloacetate, glyoxylate and hydroxypyruvate, although it generally exhibited higher catalytic efficiencies for these substrates than ECLDH. In contrast, FNLDH showed a broad substrate specificity toward relatively bulky hydrophobic 2-ketoacids, and exhibited particularly high catalytic activity toward 2-ketobutyrate and 2-ketovalerate, for which the catalytic efficiencies were comparable to that for pyruvate. FNLDH also showed relatively high activity to oxaloacetate and hydroxypyruvate, but poor activity toward glyoxylate.

**Table 1:** **Kinetic parameters for various 2-ketoacids at pH 7.0**^**a**^

	***k***_**cat**_**(s**^**−1**^**)**	***S***_**0.5**_**(mM)**	***k***_**cat**_**/*S***_**0.5**_**(s**^**−1**^ **mM**^**−1**^**)**	***K***_**i**_**(mM)**^**b**^	***n***_**H**_^**c**^
Glyoxylate					
FNLDH	11 (0.1)	18 (0.4)	0.70 (0.007)		
PALDH	880 (60)	6.8 (0.6)	130 (5)	44 (8)	
ECLDH	100 (5)	20 (2)	5.3 (0.2)		1.5 (0.09)
Pyruvate					
FNLDH	80 (0.4)	0.34 (0.007)	230 (4)		
PALDH	400 (10)	0.10 (0.009)	4,000 (300)	1.8 (0.1)	
ECLDH	410 (8)	2.6 (0.09)	160 (2)		1.4 (0.05)
2-Ketobutyrate					
FNLDH	140 (0.7)	0.31 (0.006)	440 (7)		
PALDH	100 (0.6)	0.64 (0.01)	150 (3)		
ECLDH	31 (2)	30 (4)	1.0 (0.05)		1.4 (0.08)
2-Ketovalerate					
FNLDH	140 (0.5)	0.68 (0.01)	200 (3)		
PALDH	69 (0.5)	4.0 (0.07)	18 (3)		
ECLDH	N.D.^d^	N.D.	<0.01		
2-Ketoisovalerate					
FNLDH	120 (0.7)	3.5 (0.07)	35 (0.5)		
PALDH	N.D.	N.D.	<0.4		
ECLDH	N.D.	N.D.	<0.01		
2-Ketocaproate					
FNLDH	230 (1)	2.6 (0.06)	87 (1)		
PALDH	160 (1)	5.8 (0.1)	27 (0.4)		
ECLDH	N.D.	N.D.	<0.01		
2-Ketoisocaproate					
FNLDH	200 (2)	3.8 (0.1)	53 (1)		
PALDH	39 (0.3)	12 (0.2)	3.2 (0.03)		
ECLDH	N.D.	N.D.	<0.01		
Oxaloacetate					
FNLDH	80 (1)	2.5 (0.09)	31 (0.7)	140 (20)	
PALDH	410 (4)	1.7 (0.06)	230 (6)		
ECLDH	1,100 (200)	54 (10)	21 (0.6)	5.0 (1)	
Hydroxypyruvate					
FNLDH	160 (2)	18 (0.4)	8.7 (0.07)		
PALDH	420 (10)	3.0 (0.2)	140 (3)	19 (1)	
ECLDH	50 (0.5)	12 (0.3)	4.2 (0.06)		
Phenylpyruvate					
FNLDH	120 (7)	11 (0.9)	11 (0.3)	23 (3)	
PALDH	18 (0.2)	7.1 (0.2)	2.6 (0.04)		
ECLDH	N.D.	N.D.	<0.01		
Hydroxyphenylpyruvate					
FNLDH	N.D.	N.D.	<0.02		
PALDH	N.D.	N.D.	<0.4		
ECLDH	N.D.	N.D.	<0.01		

^a^Parameters were determined as described in the ‘Materials and methods’ section, and standard deviations are shown in parentheses. ^b^The inhibition constants (*K*_i_) and ^c^Hill coefficients (*n*_H_) are shown only for the cases in which significant substrate inhibition and cooperativity were observed, respectively. ^d^N.D., not determined. The activities were too weak for determination of exact values.

### 3.4 Inhibition and activation by oxamate

**Figure 4:**
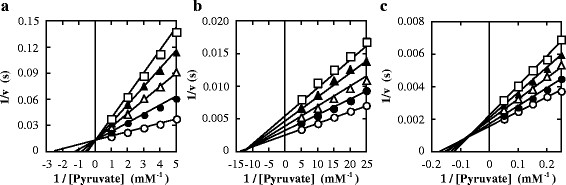
**Effects of oxamate on the catalytic reactions at pH 7.0.** The reaction velocities for FNLDH **(a)**, PALDH **(b)**, and ECLDH **(c)** were measured in 50 mM MOPS-NaOH buffer (pH 7.0) containing 0.1 mM NADH, the indicated concentrations of pyruvate and several concentrations of oxamate. The concentrations of oxamate for FNLDH were 0 mM (open circles), 10 mM (closed circles), 20 mM (open triangles), 30 mM (closed triangles), and 40 mM (open squares). The concentrations of oxamate for PALDH were 0 mM (open circles), 0.1 mM (closed circles), 0.2 mM (open triangles), 0.3 mM (closed triangles), and 0.4 mM (open squares). The concentrations of oxamate for ECLDH were 0 mM (open circles), 5 mM (closed circles), 10 mM (open triangles), 15 mM (closed triangles), and 20 mM (open squares). The reaction velocity and the concentration of pyruvate were plotted reciprocally. The data for PALDH and ECLDH were interpreted using the equation for mixed type inhibition, whereas the data for FNLDH were interpreted using the equation for competitive type inhibition. The kinetic parameters were as follows; FNLDH: *k*_cat_ = 79 ± 1 (s^−1^), *K*_m_ = 0.39 ± 0.01 (mM), and *K*_i_ = 9.4 ± 0.2 (mM). PALDH: *k*_cat_ = 410 ± 10 (s^−1^), *K*_m_ = 0.074 ± 0.006 (mM), *K*_i_ = 0.29 ± 0.04 (mM), and *K*_i_’ = 0.33 ± 0.04 (mM). ECLDH: *k*_cat_ = 670 ± 10 (s^−1^), *K*_m_ = 5.6 ± 0.3 (mM), *K*_i_ = 20 ± 2 (mM), and *K*_i_’ = 47 ± 6 (mM). GraFit ver 7.0.3 was used for non-linear regression and calculation of values. The lines were calculated with kinetic parameters.

**Figure 5:**
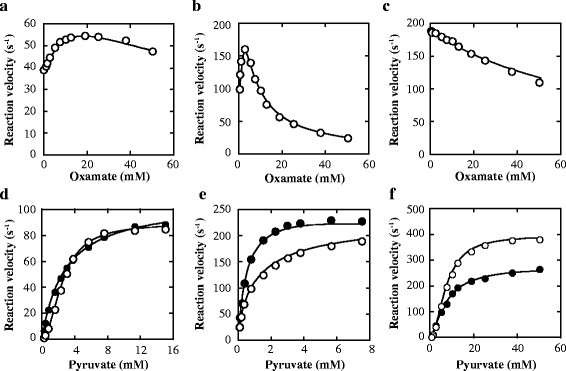
**Effects of oxamate on the catalytic reactions at pH 8.0. a-c)** The saturation curves for oxamate. The reaction velocities for FNLDH **(a)**, PALDH **(b)**, and ECLDH **(c)** were measured in 50 mM Bicine-NaOH buffer (pH 8.0) containing 0.1 mM NADH, 2.5 mM (for FNLDH), 1.2 mM (for PALDH), or 7.5 mM (for ECLDH) pyruvate, and the indicated concentration of oxamate. The data for FNLDH, PALDH, and ECLDH were interpreted using the equation for competitive-type, mix-type, and noncompetitive-type inhibition of allosteric enzyme. The kinetic parameters were as follows; FNLDH: *K*_i_ = 51 ± 10 (mM), *k*_cat_’ = 80 ± 20 (s^−1^), *n*_H_’ = 1.1 ± 0.1, and *K*_act_ = 9.9 ± 3 (mM). PALDH: *K*_i_*K*_i_’ / (*K*_i_ + *K*_i_’) = 2.0 ± 0.6 (mM), *k*_cat_’ = 470 ± 100 (s^−1^), *n*_H_’ = 1.7 ± 0.2, and *K*_act_ = 1.7 ± 0.4 (mM). ECLDH: *K*_i_ = 80 ± 3 (mM). The lines indicate the calculated saturation curves obtained with kinetic parameters. **d-f)** the saturation curves for pyruvate with or without oxamate. The reaction velocities for FNLDH **(d)**, PALDH **(e)**, and ECLDH **(f)** were measured in 50 mM Bicine-NaOH buffer (pH 8.0) in the presence of 0.1 mM NADH and the indicated concentrations of pyruvate with no effector (open circles), or 20 mM (for FNLDH), 2.5 mM (for PALDH), or 30 mM (for ECLDH) oxamate (closed circles). The lines indicate the calculated saturation curves obtained with kinetic parameters.

Oxamate, an inert pyruvate analogue, inhibited the reactions of the three enzymes at pH 7.0, where the enzymes showed no marked homotropic cooperativity, in different manners, i.e. an apparently competitive manner with pyruvate for FNLDH, and a mixed manner for PALDH and ECLDH (Figure 4). This suggests that oxamate is bound not only to the catalytic site, but also to unknown allosteric sites in PALDH and ECLDH, whereas it is bound mostly to the catalytic site in FNLDH. Oxamate apparently enhanced the reactions of FNLDH and PALDH, and exhibited the highest activating effects at 20 mM and 2.5 mM, respectively (Figure 5a, b), whereas oxamate inhibited the ECLDH reaction (Figure 5c), at pH 8.0, where the three enzymes exhibited marked homotropic cooperativity. FNLDH and PALDH showed no significant homotropic (positive or negative) cooperativity in the presence of 20 mM and 2.5 mM oxamate, respectively (Figure 5d, e). In ECLDH, the inhibition occurred in a noncompetitive manner toward pyruvate (Figure 5f), suggesting that it is mostly bound to the allosteric site of ECLDH at pH 8.0.

### 3.5 Heterotropic activation of allosteric d-LDHs

**Figure 6:**
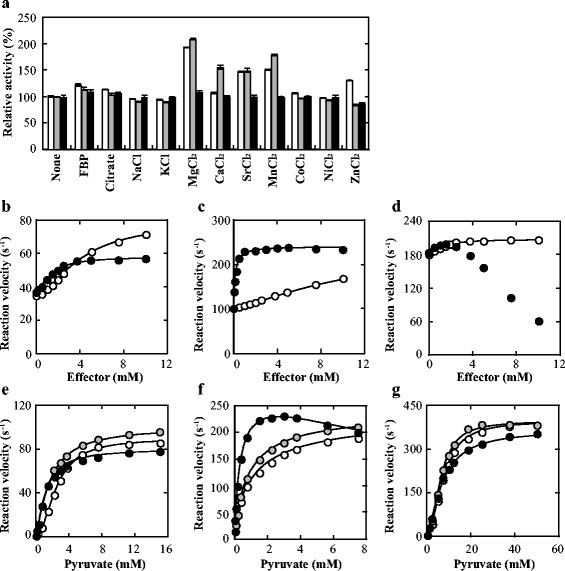
**Effects of intermediary metabolites and ions at pH 8.0. a)** Effects of intermediary metabolites and ions on FNLDH (white boxes), PALDH (grey boxes), and ECLDH (black boxes). The activities were measured in 50 mM Bicine-NaOH buffer (pH 8.0) containing 0.1 mM NADH, 2.5 mM (for FNLDH), 1.2 mM (for PALDH), or 7.5 mM (for ECLDH) pyruvate, and 1 mM indicated effectors. **b-d**) the saturation curves for FBP or MgCl_2_. The reaction velocities for FNLDH **(b)**, PALDH **(c)**, and ECLDH **(d)** were measured in 50 mM Bicine-NaOH buffer (pH 8.0) containing 0.1 mM NADH, 2.5 mM (for FNLDH), 1.2 mM (for PALDH), or 7.5 mM (for ECLDH) pyruvate, and the indicated concentrations of FBP (open circles) or MgCl_2_ (closed circles). The lines indicate the calculated saturation curves obtained with apparent kinetic parameters (Table 2). The data of MgCl_2_ saturation curve of ECLDH was not fitted with any equations used in this study. **e-g)** the saturation curves for pyruvate with or without FBP or MgCl_2_. The reaction velocities for FNLDH **(e)**, PALDH **(f)**, and ECLDH **(g)** were measured in 50 mM Bicine-NaOH buffer (pH 8.0) containing 0.1 mM NADH, the indicated concentrations of pyruvate with no effector (white circles), 10 mM (for FNLDH and PALDH) or 5 mM (for ECLDH) FBP (grey circles), and 5 mM (for FNLDH and PALDH) or 2.4 mM (for ECLDH) MgCl_2_ (black circles). The lines indicate the calculated saturation curves obtained with kinetic parameters (Table 3).

Bacterial allosteric l-LDHs are commonly activated by FBP, which usually induces drastic improvement of the substrate *S*_0.5_ values of the enzymes (Garvie [1980]). In addition, *Lactobacillus casei*l-LDH requires some divalent metal ions (e.g., Mn^2+^) (Arai et al. [2011]), and the *Thermus caldophilus* enzyme is activated also by citrate under slightly acidic conditions (Taguchi et al. [1984]). We therefore evaluated the effects of FBP, citrate and divalent metal ions at pH 8.0 in the presence of the *S*_0.5_ pyruvate. FBP and citrate (1.0 mM) slightly enhanced the catalytic reactions of the three d-LDHs, and divalent metal ions such as Mg^2+^ and Mn^2+^ more markedly enhanced the reactions of FNLDH and PALDH (Figure 6a). In the case of FNLDH, Mg^2+^ and FBP showed significant activation effects, and Mg^2+^ gave a 2.8-fold smaller *S*_0.5_ value (1.5 mM) than FBP (4.2 mM) for the enzyme activation (Figure 6 and Table 2). In the presence of 10 mM FBP or 5 mM Mg^2+^, FNLDH exhibited hyperbolic pyruvate saturation curves, the pyruvate *S*_0.5_ (*K*_m_) value being reduced by approximately 1.4-fold or twice, respectively (Figure 6, and Table 3). For PALDH, Mg^2+^ and FBP also showed activation effects, and Mg^2+^ gave a 75-fold smaller *S*_0.5_ value (0.16 mM) than FBP (12 mM) for the enzyme activation (Figure 6 and Table 2). In this case, FBP and Mg^2+^ also consistently reduced the pyruvate *S*_0.5_ value significantly, and the negative cooperativity in pyruvate binding (Figure 6, and Table 3). In the case of ECLDH, FBP and Mg^2+^ exhibited only slight activation effects on the enzyme reaction, and the latter even markedly inhibited the reaction at high concentrations (Figure 6, and Tables 2 and 3).

**Table 2:** **Apparent kinetic parameters for FBP and MgCl**_**2**_**at pH 8.0**^**a**^

	***k***_**cat**_**(s**^**−1**^**)**	***v***_**min**_**(s**^**−1**^**)**^**b**^	***S***_**0.5**_**(mM)**	***n***_**H**_^**c**^
FBP				
FNLDH	45 (1)	35 (0.6)	4.2 (0.2)	1.7 (0.05)
PALDH	150 (30)	100 (2)	12 (3)	1.3 (0.07)
ECLDH	25 (1)	180 (2)	1.3 (0.01)	1.5 (0.2)
Mg^2+^				
FNLDH	22 (0.7)	37 (0.2)	1.5 (0.1)	1.3 (0.09)
PALDH	140 (2)	100 (1)	0.16 (0.008)	1.1 (0.08)
ECLDH	N.D.^d^	N.D.	N.D.	

^a^Parameters were determined as described in the ‘Materials and methods’ section, and standard deviations are shown in parentheses. ^b^*v*_min_ is reaction velocity in the absence of FBP or Mg^2+^. ^c^Hill coefficients (*n*_H_) are shown only for the cases that exhibited significant cooperativity. ^d^N.D., not determined.

**Table 3:** **Kinetic parameters for pyruvate and NADH**^**a**^

	***k***_**cat**_**(s**^**−1**^**)**	***S***_**0.5**_**(mM)**	***k***_**cat**_**/S**_**0.5**_**(s**^**−1**^ **mM**^**−1**^**)**	***K***_**i**_**(mM)**^**b**^	***n***_**H**_^**c**^
Pyruvate					
pH 7.0					
FNLDH	80 (0.4)	0.34 (0.007)	230 (4)		
PALDH	400 (10)	0.10 (0.009)	4,000 (300)	1.8 (0.1)	
ECLDH	410 (8)	2.6 (0.09)	160 (2)		1.4 (0.05)
pH 8.0					
FNLDH	87 (1)	2.5 (0.06)	35 (0.6)		2.0 (0.1)
PALDH	240 (6)	1.2 (0.1)	200 (10)		0.77 (0.03)
ECLDH	380 (5)	7.6 (0.1)	50 (0.4)		2.2 (0.06)
pH 8.0 + FBP^d^					
FNLDH	100 (1)	1.7 (0.06)	60 (1)		1.3 (0.04)
PALDH	240 (4)	0.85 (0.04)	280 (6)		0.9 (0.02)
ECLDH	400 (5)	6.5 (0.1)	61 (1)		2.1 (0.09)
pH 8.0 + Mg^+2e^					
FNLDH	80 (1)	1.2 (0.04)	65 (1)		1.4 (0.05)
PALDH	290 (3)	0.36 (0.01)	820 (20)	20 (0.8)	
ECLDH	370 (6)	7.2 (0.2)	51 (1)		1.5 (0.07)
NADH					
pH 7.0					
FNLDH	98 (0.7)	0.022 (0.0005)	4,400 (80)		
PALDH	310 (2)	0.019 (0.0005)	17,000 (400)		
ECLDH	640 (20)	0.055 (0.004)	12,000 (500)		
pH 8.0					
FNLDH	90 (1)	0.036 (0.001)	2,500 (30)		1.6 (0.07)
PALDH	270 (2)	0.035 (0.0007)	7,700 (100)		
ECLDH	710 (8)	0.088 (0.002)	8,100 (100)		
pH 8.0 + FBP^d^					
FNLDH	80 (1)	0.028 (0.001)	2,800 (70)		1.5 (0.07)
PALDH	270 (2)	0.035 (0.001)	7,800 (100)		
ECLDH	730 (10)	0.092 (0.003)	8,000 (100)		
pH 8.0 + Mg^+2e^					
FNLDH	110 (2)	0.031 (0.001)	3,700 (70)		1.3 (0.05)
PALDH	270 (2)	0.035 (0.0009)	7,700 (100)		
ECLDH	690 (9)	0.090 (0.002)	7,700 (100)		

^a^Parameters were determined as described in the ‘Materials and methods’ section, and standard deviations are shown in parentheses. ^b^The inhibition constants (*K*_i_) are shown only for the cases that exhibited significant substrate inhibition. ^c^Hill coefficients (*n*_H_) are shown only for the cases that exhibited significant cooperativity. ^d^The concentrations of FBP for ECLDH, FNLDH, and PALDH were 5 mM, 10 mM, and 10 mM, respectively. ^e^The concentrations of Mg^2+^ for ECLDH, FNLDH, and PALDH were 2.4 mM, 5 mM, and 5 mM, respectively.

### 3.6 Kinetic profiles for NADH

The apparent kinetic parameters for NADH were determined at the saturation concentration of pyruvate at pH 7.0 and 8.0 (Table 3). FNLDH and PALDH exhibited virtually the same apparent NADH *S*_0.5_ value (about 0.035 mM), which was approximately twice smaller than that of ECLDH. Since the pyruvate *S*_0.5_ values were determined at 0.1 mM NADH (Figure 3 and Table 3), these values might be larger than the real values because of an insufficient concentration of NADH, particularly in the case of ECLDH. PALDH and ECLDH exhibited hyperbolic NADH saturation curves independently of pH, FBP or Mg^2+^. On the other hand, FNLDH exhibited a sigmoidal one at pH 8.0, and exhibited an *n*_H_ value of 1.6, which was slightly reduced by FBP or Mg^2+^.

## 4 Discussion

The characteristics of FNLDH, PALDH and ECLDH demonstrate the high variety of the functions of bacterial d-LDHs. Of the three enzymes, PALDH exhibits a markedly smaller pyruvate *S*_0.5_ value than ECLDH, and a larger *k*_cat_ value than FNLDH at pH 7.0 (Table 3). Although the crucial role of PALDH in the metabolism of *P. aeruginosa* remains uncertain, the enzyme is likely involved in regulation of the NAD^+^/NADH ratio within the cells like the heart-type l-LDH isozymes of aerobic tissues (Holbrook et al. [1975]). On the other hand, ECLDH with the large *S*_0.5_ is likely favorable for facultative anaerobes such as *E. coli*, in which the pyruvate level within the cells dynamically changes between anaerobic and aerobic conditions like the muscle-type l-LDH isozymes (Holbrook et al. [1975]). FNLDH exhibits a small pyruvate *S*_0.5_ value although *F. nucleatum* is obligate anaerobe. In *F. nucleatum* cells, pyruvate is likely consumed mostly through pathways other than lactate fermentation, such as butyrate fermentation (Kapatral et al. [2002]), and therefore pyruvate within the cells may be maintained at low concentrations. It is particularly notable that FNLDH prefers relatively bulky hydrophobic 2-ketoacids, such as 2-ketobutyrate and 2-ketovalerate, which give apparently equivalent or even higher *k*_cat_/*K*_m_ value than pyruvate, unlike in the cases of the other two enzymes (Table 1). This substrate preference suggests that FNLDH might be used for not only pyruvate reduction but also the reduction or oxidation of other bulky 2-ketoacid or 2-hydroxy acids, respectively, although the actual physiological substrates of the enzyme remains uncertain.

FNLDH apparently has a size-preference for 2-ketoacid substrates intermediate between those of conventional d-LDH and d-2-hydroxyisocaproate dehydrogenase (Hummel et al. [1985]). Like usual d-LDHs, nevertheless, FNLDH has aromatic residues, Phe52 and Tyr299 (residue numbers are according to d-LDH from *Lactobacillus pentosus*), which are proposed to play key roles in the substrate recognition in the binding pocket for the substrate C3 side chains (Tokuda et al. [2003]; Ishikura et al. [2005]). It is also noteworthy that FNLDH exhibits relatively high activity toward 2-ketoisovalerate since little is known as to an enzyme that exhibits high catalytic activity toward C3-branched 2-ketoacid substrates in the d-LDH-related d-HydDH family. It is known that d-mandelate dehydrogenase from *Enterococcus faecalis* exhibits high catalytic activity toward hydrophobic C3-branched ketoacids such as 2-ketoisovalerate and benzoyl formate (Tamura et al. [2002]). Nevertheless, this enzyme belongs to another d-HydDH family, the 2-ketopantate reductase-related family, which is evolutionally separate from the d-LDH-related family (Wada et al. [2008]; Miyanaga et al. [2013]). Hence, FNLDH is the first case of a d-LDH-related d-HydDH that exhibits relatively high catalytic activity toward C3-branched 2-ketoacid substrates and may be useful for enzymatic production of C3-branched d-2-hydroxyacids.

For FNLDH, oxamate inhibits the reaction competitively with pyruvate at pH 7.0, and substitutes for the role of pyruvate in the homotropic enzyme activation at pH 8.0, converting the sigmoidal saturation curve for pyruvate to a hyperbolic one (Figure 5d). These effects indicate that oxamate simply competes with pyruvate for a common binding site, i.e., the catalytic site in FNLDH. It is likely that PALDH binds oxamate at both the catalytic and allosteric sites, since oxamate inhibits the reaction in a mixed manner at pH 7.0 (Figure 4), as in the case of ECLDH, but stimulates the reaction at low concentration at pH 8.0, where oxamate likely exhibits an activation effect mostly through binding to the catalytic site and inhibitory effects mostly through binding to the unknown allosteric site (Figure 5b,e). In the case of ECLDH, it was suggested previously that oxamate is likely bound mostly to a binding site other than the catalytic site (Tarmy and Kaplan [1968]), this being consistent with the results of this study (Figure 4c). These results indicate that the three enzymes differ in their allosteric behaviors, and specificities to allosteric effectors, although the actual allosteric binding sites or physiological effectors of these enzymes remain uncertain.

FNLDH and PALDH are the first cases of d-LDHs that are activated by FBP, which is a common activator for bacterial allosteric l-LDHs (Garvie [1980]). It is nevertheless unlikely that FBP is really the specific activator of the two d-LDHs, since the two enzymes require apparently higher concentrations of FBP than the physiological one for their activation (Figure 6b,c and Table 2). On the other hand, the Mg^2+^ ion is more effective than FBP as to activation of the two d-LDHs, particularly PALDH (Figure 6, and Tables 2 and 3). It is, however, also unlikely that the Mg^2+^ ion is the specific activator of these enzymes, since some other divalent metal ions such as Sr^2+^ and Mn^2+^ also improve their reactions (Figure 6a). These two enzymes thus appear to be activated through the comprehensive effects of several activators with low specificities, physiologically, although it is possible that they have unknown activators with higher specificities. In the case of ECLDH, FBP and Mg^2+^ only slightly enhance the catalytic reaction, and the latter even markedly reduces the reaction at higher concentrations (Figure 6d,g and Table 3). We have evaluated other possible effectors, such as l-alanine, l-glutamate and l-aspartate, for this enzyme, but have not found out any potential activator for ECLDH (data not shown). This implies that ECLDH is only negatively controlled under alkaline conditions.

The allosteric and tetrameric d-PgDHs from *E. coli* and *M. tuberculosis* were extensively studied as to their structure-function relationship (Tobey and Grant [1986]; Grant [1989]; Schuller et al. [1995]; Dey et al. [2005]; Grant [2012]). The d-PgDHs have *V*-type regulatory mechanisms, in which l-serine inhibits the enzyme reaction in a non-competitive manner, whereas the three d-LDHs apparently exhibit *K*-type rather than *V*-type regulation. These d-PgDHs have additional functional domain(s), ACT domain and ASB domain, which undergo not only interactions with allosteric effector, l-serine, but also inter-subunit interactions with each other for the construction of tetrameric structure. In contrast, d-LDHs have no additional protein domain. The three d-LDHs from Gram-negative bacteria thus greatly differ from these d-PgDHs in both regulatory mechanism and protein structure.

It is reported that bacterial allosteric l-LDHs consistently undergo the Monod-Wyman-Changeux-type (MWC-type: pre-existing type) (Monod et al. [1965]; Iwata et al. [1994]; Arai et al. [2010]) allosteric transition, where the active (R) state with high affinity to pyruvate and FBP, and the inactive (T) state with low affinity. However, such drastic structural changes appear to be unnecessary for the three d-LDHs, which exhibit apparently much smaller changes in catalytic activity due to the allosteric transition than the l-LDHs. In fact, the MWC model is not available for the case of PALDH, which shows negative homotropic cooperativity. In addition, FNLDH shows significant positive homotropic cooperativity also for NADH binding, which is less affected by FBP or Mg^2+^ than pyruvate binding (Table 3), suggesting that FNLDH changes in its structure through two steps, the NADH binding and following pyruvate binding steps, in the allosteric transition. The *Lactobacillus*d-LDHs consistently have dimeric structure, whereas the three d-LDHs from Gram-negative bacteria have tetrameric structure. Thus, tetramerization might be correlated with the allostery of d-LDHs. It is highly desirable to determine the 3D-structures of these d-LDHs.

## Abbreviations

d-LDH: NAD-dependent d-lactate dehydrogenase

l-LDH: l-lactate dehydrogenase

d-HydDH: d-2-hydroxyacid dehydrogenase

d-PgDH: d-3-phosphoglycerate dehydrogenase

ECLDH: d-LDH from *Escherichia coli*

FNLDH: d-LDH from *Fusobacterium nucleatum*

PALDH: d-LDH from *Pseudomonas aeruginosa*

FBP: Fructose 1,6-bisphosphate

MWC: Monod-Wyman-Changeux

## Competing interests

The authors declare that they have no competing interests.

## Authors’ contributions

NF designed the study, carried out most of the biochemical studies and drafted the manuscript. AM designed the study and helped to draft the manuscript. MT participated in the kinetics analysis of PALDH. MN participated in the design of the study and the kinetics analysis, and helped to draft the manuscript. HT conceived and designed the study, and helped to draft the manuscript. All authors read and approved the final manuscript.
